# The impact of integrase inhibitors on steatosis and fibrosis biomarkers in persons with HIV naïve to antiretroviral therapy

**DOI:** 10.1186/s12879-023-08530-3

**Published:** 2023-08-24

**Authors:** Sara Rodrigues Fernandes, Ana Rita Leite, Rita Lino, André Rodrigues Guimarães, Carmela Pineiro, Rosário Serrão, Paula Freitas

**Affiliations:** 1 Faculdade de Medicina, Universidade do Porto, Porto, Portugal; 2 Serviço de Endocrinologia, Diabetes e Metabolismo do Centro Hospitalar Universitário São João, Faculdade de Medicina, Universidade do Porto, Porto, Portugal; 3 Departamento de Doenças Infeciosas do Centro Hospitalar Universitário São João, Porto, Portugal; 4 Serviço de Endocrinologia, Diabetes e Metabolismo do Centro Hospitalar Universitário São João, Faculdade de Medicina, Investigação e Inovação em Saúde (i3s), Universidade do Porto, Porto, Portugal

**Keywords:** HIV, Integrase strand transfer inhibitors, Non-alcoholic fatty liver disease, Steatosis, Liver fibrosis

## Abstract

**Background:**

Non-alcoholic Fatty Liver Disease (NAFLD) has a high prevalence among persons with HIV infection. Since Integrase Strand Transfer Inhibitors (INSTIs) are used worldwide and have been associated with weight gain, we must determine their effect in the development of NAFLD and Non-alcoholic Steatohepatitis (NASH) in these patients. The aim of this study was to explore the impact of INSTIs on variation of liver steatosis and fibrosis in the ART-naïve person with HIV, using Hepatic Steatosis Index (HSI), Fibrosis-4 Index (FIB-4), BARD score and NAFLD Fibrosis Score (NFS).

**Methods:**

We performed a monocentric, retrospective cohort study in ART-naïve persons with HIV that initiated INSTI based regimens between December 2019 and January 2022. Data was collected at baseline, 6 and 12 months after initiation. Demographic, clinical and laboratory characteristics, hepatic steatosis, and fibrosis scores were compared between baseline and last visit at 12 months. Linear regression models were performed to analyse the associations between analytical data at baseline and hepatic scores variation during the 12 months of treatment. Models were performed unadjusted and adjusted for age and sex.

**Results:**

99 patients were included in our study. 82% were male and median age was 36 years. We observed a significant increase in body mass index (BMI), HDL, platelet count, albumin, and creatinine and a significant decrease in AST levels. HSI showed no statistically significant differences during follow-up (p = 0.114). We observed a significant decrease in FIB-4 (p = 0.007) and NFS (p = 0.002). BARD score showed a significant increase (p = 0.006). The linear regression model demonstrated a significant negative association between baseline HIV RNA and FIB-4 change (β= -0.08, 95% CI [-0.16 to -0.00], p = 0.045), suggesting that higher HIV RNA loads at baseline were associated with a greater decrease in FIB-4.

**Conclusion:**

INSTIs seem to have no impact on hepatic steatosis, even though they were associated with a significant increase in BMI. This might be explained by the direct effect of a dolutegravir-containing regimen and/or by the “return-to-health effect” observed with ART initiation. Furthermore, INSTIs were associated with a reduction in risk of liver fibrosis in ART-naïve persons with HIV, possibly due to their effect on viral suppression.

**Supplementary Information:**

The online version contains supplementary material available at 10.1186/s12879-023-08530-3.

## Background

Improvements in Human Immunodeficiency Virus (HIV) infection treatment has shifted the priorities in the clinical care of patients with this infection. Due to the increased access to combined Antiretroviral Therapy (ART), mortality amongst persons with HIV has declined and life expectancy has been approaching that of the general population. Even though it remains the leading cause of death in this group of patients, Acquired Immunodeficiency Syndrome (AIDS)-related mortality has decreased, hence increasing the importance of non-AIDS related morbidities, such as non-AIDS cancers, liver disease, cardiovascular diseases, and stroke [1, 2].

Non-alcoholic Fatty Liver Disease (NAFLD) is characterized by evidence of hepatic steatosis, without secondary causes for hepatic fat accumulation, and is related to metabolic comorbidities. NAFLD is divided into two categories, Non-alcoholic fatty liver (NAFL) and Non-alcoholic Steatohepatitis (NASH). NAFL is defined as the presence of steatosis in ≥ 5% of hepatocytes without hepatocyte ballooning. NASH is defined as the presence of steatosis in ≥ 5% of hepatocytes and inflammation with hepatocyte injury, associated or not to fibrosis [3].

Although the true prevalence of NAFLD in persons with HIV is still unknown, Maurice et al. showed a prevalence of NAFLD and NASH, in these patients, of 35% and 42%, respectively [4]. According to Vodkin et al., there is a higher proportion of NASH and features of more severe liver injury in patients with HIV-associated NAFLD, when compared with patients with primary NAFLD, despite having similar metabolic characteristics [5].

Multiple risk factors have been associated with the development of NAFLD in persons with HIV. These include factors that also have an association with NAFLD in the general population, such as male sex, obesity, hypertriglyceridemia, and insulin resistance. However, factors associated with HIV itself, such as lipodystrophy and ART, contribute to the disease as well [6, 7].

Previous studies have suggested the contribution of ART in the development of hepatic steatosis, due to its metabolic side effects [8]. In particular, various HIV protease inhibitors (PIs) have been associated with higher levels of insulin resistance. Most PIs, some Non-Nucleoside Reverse-Transcriptase Inhibitors (NNRTIs) such as efavirenz and some Nucleoside/nucleotide Reverse-Transcriptase Inhibitors (NRTIs) such as abacavir have been related to dyslipidemia. Stavudine and didanosine have been shown to induce mitochondrial toxicity, which also contributes to the development of NASH [9].

Bischoff et al., demonstrated that the use of Integrase Strand Transfer Inhibitors (INSTIs) and/or Tenofovir-alafenamid (TAF) contributes to the occurrence of hepatic steatosis and progression to NASH, in the context of increased body weight [10].

Liver biopsy is the gold standard for identifying both NASH and NAFLD. However, it has various limitations, as it is an invasive procedure with high costs, low acceptability, and sampling variability. Therefore, multiple non-invasive strategies have been studied and developed, as alternatives to this technique, including blood biomarkers and imaging techniques [11]. Scores based on blood biomarkers available to diagnose or grade steatosis include the Hepatic Steatosis Index (HSI), and to stage fibrosis include NAFLD Fibrosis Score (NFS) and BARD, which are more specific of NAFLD, and Aspartate Transaminase (AST)/Alanine Transaminase (ALT) Ratio and Fibrosis-4 Index (FIB-4), which have been developed in the context of hepatitis C [12].

According to the European Association for the Study of the Liver-European Association for the Study of Diabetes-European Association for the Study of Obesity (EASL-EASD-EASO) Clinical Practice Guidelines for the management of non-alcoholic fatty liver disease, NFS, FIB-4, Enhanced Liver Fibrosis (ELF) or FibroTest calculation should be performed in every NAFLD patient to exclude significant fibrosis. If fibrosis is not excluded, then transient elastography should be performed. Only if this exam confirms significant fibrosis, should liver biopsy be done in order to establish the final diagnosis [13].

Currently, INSTIs are recommended worldwide as first line treatment in HIV infection [14]. With the growing number of patients under this treatment and the high prevalence of liver disease in persons with HIV, it becomes essential to determine the effect of these drugs in the development of NAFLD and liver fibrosis.

Therefore, we performed a retrospective cohort study with the aim of evaluating the impact of INSTIs on variation of steatosis and fibrosis biomarkers, using HSI, FIB-4, BARD and NFS indexes, in persons with HIV infection.

## Methods

### Subjects

We performed an observational monocentric, retrospective cohort study in persons with HIV followed at the Infectious Diseases Outpatient Clinic of Centro Hospitalar Universitário de São João. This study included all treatment-naïve adults (age ≥ 18 years) that initiated an INSTI based regimen between December 2019 and January 2022 and maintained it during at least 12 months. Exclusion criteria were Hepatitis C Virus (HCV) and/or Hepatitis B Virus (HBV) infection, determined by HCV antibody testing and HBV surface antigen positivity, pregnancy at the beginning or during follow-up and excessive alcohol use, based on the self-reported alcohol consumption by the patients. This study was approved by Comissão de Ética para a Saúde do Centro Hospitalar Universitário de São João and the requirement for a signed informed consent was waived.

### Clinical assessment

For each patient the following information was collected: demographic data (age, sex), clinical comorbidities, such as Diabetes Mellitus (DM) and smoking history, time since HIV diagnosis, HIV infection risk factors, duration of ART, ART regimen and the degree of the infection. We used the “Centers for Disease Control and Prevention” (CDC) criteria for classifying the degree of the infection [15]. Diabetes Mellitus diagnosis was determined using a combination of diagnostic code and use of antidiabetic medication. Weight and height were measured in routine consultation at baseline, before starting ART, and during follow-up. Body Mass Index (BMI) was calculated automatically using the formula: weight (kg) / height (m^2^). These data were collected through clinical records stored at the hospital’s electronic platform.

### Laboratory analysis

Serum samples were tested at baseline, before starting ART (T0), and six months (T6) and twelve months (T12) after initiating ART. CD4^+^ T cell count in cells/mm^3^, type 1 HIV Ribonucleic Acid (RNA) in copies/mL, platelet count in 10^3^/µL, albumin in g/L, AST in U/L, ALT in U/L, total bilirubin in mg/dL, total cholesterol in mg/dL, High-density Lipoprotein (HDL) cholesterol in mg/dL, Low-density Lipoprotein (LDL) cholesterol in mg/dL, Triglycerides (TG) in mg/dL, fasting glucose in mg/dL, creatinine in mg/dL, uric acid in mg/dL, and C Reactive Protein (CRP) levels in mg/dL were retrieved from clinical records through the hospital’s electronic platform.

### Hepatic steatosis and fibrosis evaluation

The HSI values were calculated automatically using the formula: 8 x (ALT/AST ratio) + Body Mass Index (BMI) (+ 2, if female; +2, if diabetes mellitus). The categories considered were NAFLD ruled out with HSI < 30.0 and NAFLD detected with HSI > 36.0 [16].

The FIB-4 values were calculated automatically using the formula: age (years) × AST [U/l] / (platelets [10^9^/l] ×$\surd$ (ALT [U/l])). FIB-4 < 1.45 was considered as no or moderate fibrosis (F0-F1-F2-F3), and FIB-4 > 3.25 was considered as extensive fibrosis or cirrhosis (F4-F5-F6) (in the ISHAK classification of fibrosis) [17].

The BARD score was calculated as BMI ≥ 28 kg/m2 (1 point) + AST/ALT ratio ≥ 0.8 (2 points) + presence of diabetes (1 point). The categories considered were low risk of advanced fibrosis (0–1 score) or high risk of advanced fibrosis (2–4 score) [18].

The NFS values were calculated automatically using the formula: -1.675 + (0.037 x age [years]) + (0.094 x BMI [kg/m2]) + (1.13 x IFG/diabetes [yes = 1, no = 0]) + (0.99 x AST/ALT ratio) – (0.013 x platelet count [×10^9^/L]) – (0.66 x albumin [g/dl]). We divided the individuals in categories based on NFS score as low risk of advanced fibrosis with NFS<-1,455, intermediate risk with NFS between − 1,455 and 0,672 and high risk with NFS > 0,672 [12].

### Statistical analysis

Demographic, clinical and laboratory characteristics and hepatic steatosis and fibrosis scores were compared between baseline and last visit at 12 months. Categorical variables were presented as absolute and relative frequencies. Continuous variables were expressed as means (standard deviation), if normally distributed, or as median (25th to 75th percentile), if non-normally distributed. Variables with skewed distribution were transformed to their natural logarithm.

Persons with missing baseline or follow-up data for the variables needed to calculate each score were excluded from the analysis of the respective score.

Differences in continuous variables between baseline and the last visit were assessed using paired t-test or Wilcoxon test, according to the distribution of the variables. McNemar test was used for categorical data.

Linear regression models were performed to analyse the associations between analytical data at baseline and the hepatic scores variation during the 12 months of treatment. Regression models were performed unadjusted and adjusted for age [19] and sex [20].

The statistical analysis was performed using SPSS version 27.0 (IBM Corporation, Armonk, NY). Two-sided *p* values < 0.05 were considered significant.

The manuscript was prepared in adherence to the STROBE guidelines for cohort studies [21].

## Results

### Characteristics of the Study Population

**Fig. 1:**
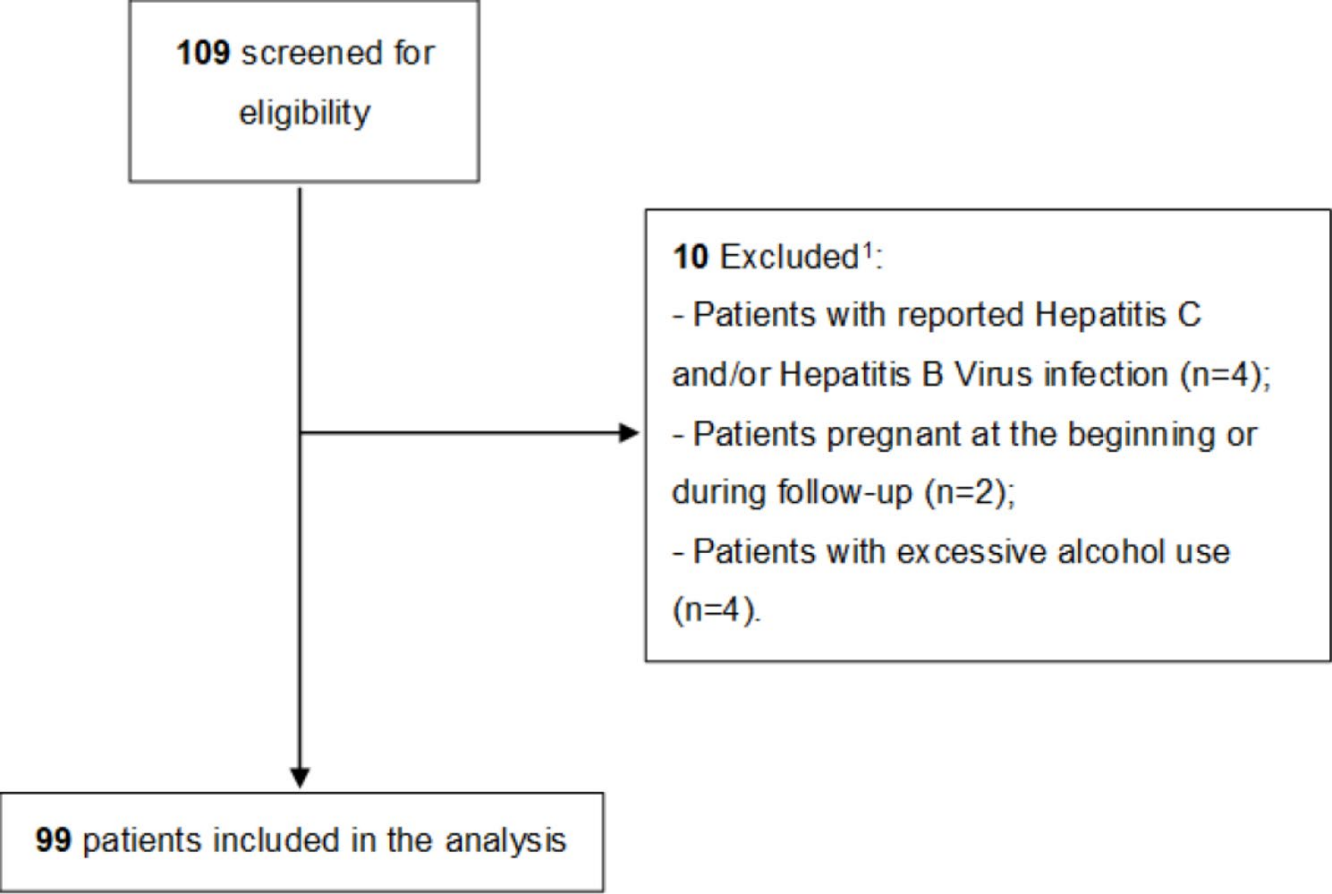
Flow diagram of the patients’ selection. ^1^Patients could meet more than 1 exclusion criteria

Overall, as demonstrated in Fig. 1, 99 patients were included in our analysis, both at baseline and through follow-up, until last visit at 12 months. 82% were male, and the median age was 36 years (28 to 50). (Table 1) The most frequent routes of transmission were men who have sex with men (60.4%) and heterosexual contact (29.7%). 39% of patients had a nadir CD4 cell count < 200/ µL and 17.2% were diagnosed as having HIV stage C.

**Table 1:** Comparison of baseline and last visit characteristics of the study population

Parameter	Baseline (n = 99)	Last Visit (n = 99)	P
Male	81 (81.8)		
Age, years	36 (28;50)	37 (29;51)	
Smoker	39 (39.4)		
BMI, kg/m^2^	23.74 (3.74)	24.61 (3.99)	**< 0.001**
25 to < 30	19 (27.94)	21 (30.88)	**0.009**
≥30	4 (5.88)	9 (13.24)	
HIV-related parameters			
HIV RNA, 10^4^ copies/mL	9.21 (3.18;25.10)	0.00 (0.00;0.03)	**< 0.001**
HIV RNA (< 50)	0	80 (79.20)	
CD4 cell count, cells/µL	259 (102;450)	539 (296;782)	**< 0.001**
HIV risk factor			
Injecting drug user	1 (0.99)		
Homosexual contact	61 (60.39)		
Heterosexual contact	30 (29.70)		
CDC stage			
A	61 (61.62)		
B	21 (21.21)		
C	17 (17.17)		
ART Regimen			
TDF/FTC + DTG	36 (36.36)		
ABC/3TC/DTG	36 (36.36)		
3TC/DTG	22 (22.22)		
FTC/TAF/BIC	4 (4.04)		
TAF/FTC + DTG	1 (1.01)		
Analytical parameters			
Fasting Plasma Glucose, mg/dL	88.00 (80.00;94.00)	89.00 (83.00;100.00)	0.590
Triglycerides, mg/dl	97.50 (72.50;130.75)	90.00 (72.00;137.00)	0.773
Total cholesterol, mg/dl	156.79 (43.74)	170.67 (45.72)	0.112
HDL, mg/dl	40.23 (12.87)	48.59 (13.19)	**< 0.001**
LDL, mg/dl	100.87 (31.84)	107.09 (32.67)	0.421
AST, U/L	26.00 (21.00;33.25)	24.00 (20.00;29.00)	**0.019**
ALT, U/L	22.00 (14.00;34.25)	19.00 (15.00;28.00)	0.115
Total bilirubin, mg/dL	0.60 (0.22)	0.59 (0.48;0.77)	0.076
Platelets, 10^3^/µL	208.61 (78.46)	234.55 (61.02)	**< 0.001**
Albumin, g/L	39.51 (7.29)	43.20 (40.80;45.10)	**< 0.001**
Creatinine, mg/dL	0.78 (0.65;0.89)	0.96 (0.26)	**< 0.001**
Uric acid, mg/dL	5.60 (4.70;6.35)	5.70 (4.80;6.40)	0.920
CRP, mg/L	4.15 (1.73;19.50)	2.60 (1.40;30.60)	0.515
Hepatic Fibrosis and Steatosis Scores			
HSI score	31.30 (26.78;34.82)	31.48 (28.21;36.37)	0.114
<30	31 (46.27)	26 (38.24)	0.388
30 to 36	23 (34.33)	25 (36.76)	1.000
>36	13 (19.40)	17 (25.00)	0.453
FIB-4 score	1.02 (0.64;1.40)	0.79 (0.60; 1.20)	**0.007**
<1.45	71 (76.34)	78 (82.98)	0.146
1.45 to 3.25	18 (19.35)	13 (13.83)	0.302
>3.25	4 (4.30)	3 (3.19)	1.000
BARD score	1.82 (0.85)	2.09 (0.73)	**0.006**
0	9 (13.43)	4 (5.88)	0.070
1	2 (2.99)	1 (1.47)	1.000
2	50 (74.63)	50 (73.53)	1.000
3	4 (5.97)	11 (16.18)	**0.016**
4	2 (2.99)	2 (2.94)	1.000
NFS score	-1.95 (-3.25; -0.75)	-2.15 (-3.29; -1.16)	**0.002**
<-1.455	38 (63.33)	45 (66.22)	0.146
-1.455 to 0.672	18 (30.00)	20 (29.41)	0.227
>0.672	4 (6.67)	2 (2.94)	1.000

Data are shown as mean (standard deviation), median (interquartile range) or n (%). P values were obtained using paired samples t-test, Wilcoxon test or McNemar test where appropriate. Statistical significance was set for a value of p < 0.05. In bold: p < 0.05

Abbreviations: 3TC, lamivudine; ABC, Abacavir, ALT, alanine aminotransferase; AST, aspartate aminotransferase; BIC, bictegravir; BMI, body mass index; CRP, C-reactive protein; DTG, dolutegravir; FIB-4, fibrosis-4; FTC, emtricitabine; HDL, high-density lipoprotein; HSI, hepatic steatosis score; HIV, human immunodeficiency virus; LDL, low-density lipoprotein; NFS, NAFLD fibrosis score; RNA, ribonucleic acid; TAF, tenofovir alafenamide; TDF, tenofovir disoproxil fumarate

We were able to calculate BMI at baseline and/or at last visit only in 68 patients, due to weight and height data availability. At baseline, overweight, defined by a BMI of at least 25 and less than 30 kg/m^2^, was observed in 19 (27.9%) patients, and obesity, defined by a BMI of at least 30 kg/m^2^, was observed in 4 (5.9%).

We observed a significant increase in BMI, HDL, platelet count, albumin, and creatinine during follow-up. Furthermore, we observed a significant decrease in AST levels.

### Hepatic fibrosis and steatosis scores

We were able to calculate the BARD and HSI scores on either T0 or T12 in 68 patients and on both visits in 59 patients. We were able to calculate the NFS score in 67 patients on at least one of the visits and in 50 patients on both T0 and T12. We were able to calculate the FIB-4 score in 94 patients on at least one of the visits and in 92 patients on both visits. We compared the baseline characteristics of the sample that had all the hepatic steatosis and fibrosis scores available in the first visit (n = 59) with the baseline characteristics of the individuals without at least one of these scores (n = 40) and observed that the characteristics of the individuals were similar between these groups and similar to the whole cohort (see Additional file 1).

The median HSI values were 31.30 (26.78 to 34.82) at baseline and 31.48 (28.21 to 36.37) at the last visit, showing no statistically significant differences (p = 0.114). The median difference in HSI score between baseline and last visit was 0.56 (-1.33 to 2.30). HSI scores < 30, ruling out the presence of NAFLD, were observed in 31 (46.27%) and 26 (38.24%) of patients at baseline and last visit, respectively. HSI values > 36, indicating presence of NAFLD, were observed in 13 (19.40%) and 17 (25.00%) of patients at baseline and last visit, respectively.

The median FIB-4 values were 1.02 (0.64 to 1.40) at baseline and 0.79 (0.60 to 1.20) at the last visit, showing a significant decrease (p = 0.007). The median difference in FIB-4 values between baseline and last visit was − 0.058 (-0.357 to 0.097). FIB-4 values < 1.45, indicating none or moderate fibrosis, were observed in 71 (76.34%) and 78 (82.98%) of patients at baseline and last visit, respectively. FIB-4 values > 3.25, indicating extended fibrosis or cirrhosis, were observed in 4 (4.30%) and 3 (3.19%) of patients at baseline and last visit, respectively.

The mean of BARD scores was 1.82 (0.85) at baseline and 2.09 (0.73) at the last visit, showing a significant increase of this score during follow-up (p = 0.006). The mean difference in BARD values between baseline and last visit was 0.37 (0.93). Eleven (16.4%) and 5 (7.4%) patients had BARD scores of either 0 or 1, representing a low risk for advanced fibrosis, at baseline and last visit, respectively. BARD scores between 2 and 4, representing a high risk of advanced fibrosis, were observed in 56 (83.6%) and 63 (92.7%) patients at baseline and last visit, respectively. However, only 13 (22%) patients had a different BARD score value between baseline and last visit. 46 (78%) patients showed no alteration in BARD score.

The median NFS values were − 1.95 (-3.35 to -0.75) at baseline and − 2.15 (-3.29 to -1.16) at the last visit, displaying a significant decrease in this score (p = 0.002). The median difference in NFS values was − 0.42 (-0.93 to 0.18) between baseline and last visit. NFS scores <-1.455, indicating low risk of advanced fibrosis, were observed in 38 (63.3%) and 45 (66.2%) of patients at baseline and last visit, respectively. NFS values between − 1.455 and 0.672, representing intermediate risk, were found in 18 (30.0%) and 20 (29.4%) patients at baseline and last visit, respectively. NFS values > 0.672, indicating high risk of advanced fibrosis, were observed in 4 (6.67%) and 2 (2.94%) patients at baseline and last visit, respectively.

**Fig. 2:**
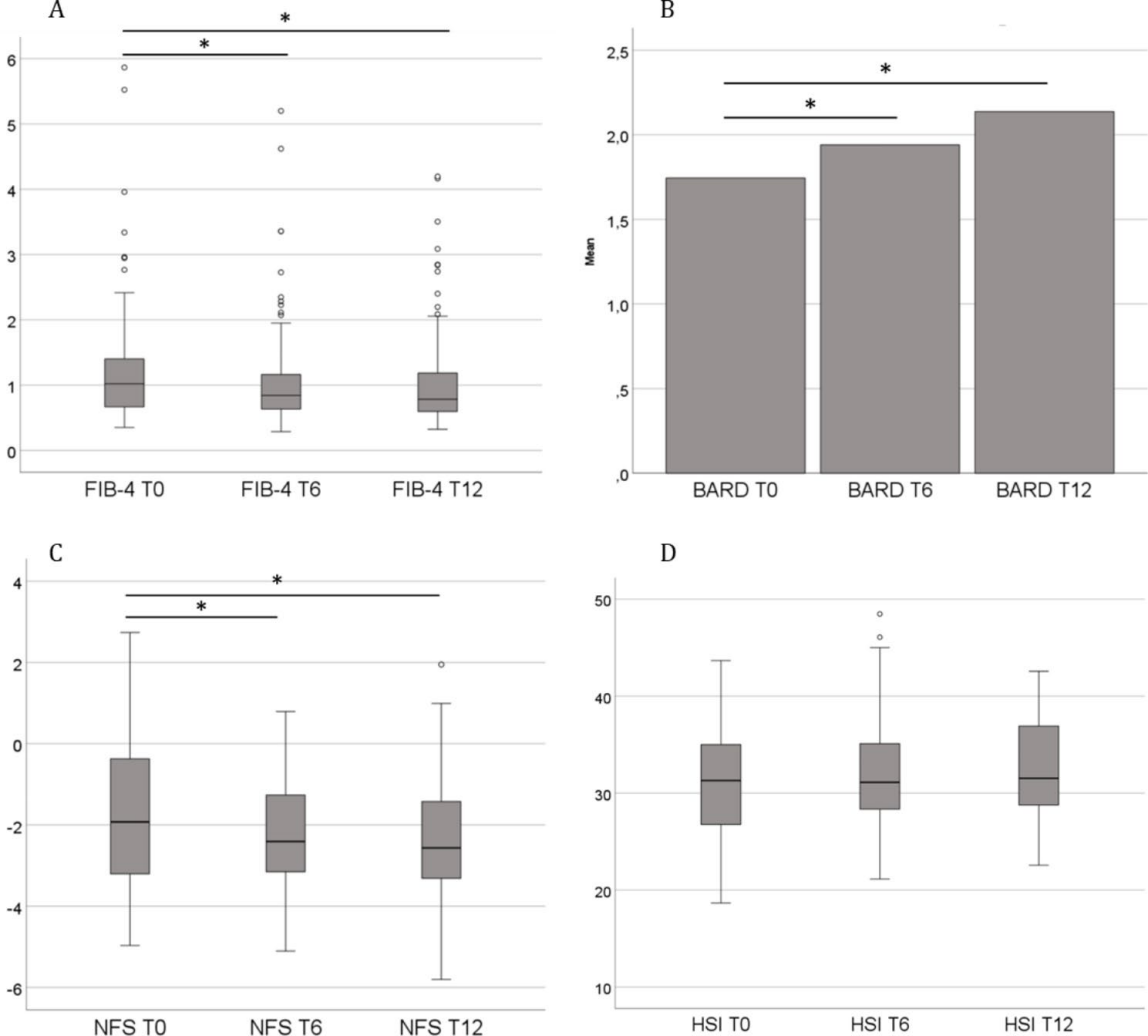
Boxplots and bar charts of liver steatosis and fibrosis scores at baseline, and 6 and 12 months after initiation of treatment with Integrase Strand Transfer Inhibitors.**A**, Box-plot of FIB-4 values at baseline (T0), 6 months (T6) and 12 months (T12) of follow-up; **B**, Bar chart of mean BARD values at T0, T6 and T12; **C**, Box-plot of NFS values at T0, T6 and T12; **D**, Box-plot of HSI values at T0, T6 and T12. * p < 0.05. Abbreviations: FIB-4, Fibrosis-4; HSI, Hepatic Steatosis Index; NFS, NAFLD Fibrosis Score.

In Fig. 2, we show a decrease in FIB-4 and NFS throughout time, at baseline, 6 and 12 months, and an increase in BARD. HSI did not vary over time.

### Analytical predictors of changes in hepatic fibrosis scores

In the unadjusted linear regression model (Table 2), there was a significant negative association between baseline HIV RNA and FIB-4 change, suggesting that higher HIV RNA loads at baseline are associated with a decrease in FIB-4 (β=-0.08 [-0.16 to 0.00]; p = 0.045). After adjusting for age and sex, this association was no longer significant, although a trend for a negative association was found (β=-0.08 [-0.16 to 0.00]; p = 0.062).

**Table 2:** Associations between analytical variables at baseline and changes in hepatic fibrosis scores

	FIB-4		BARD		NFS	
	β (95% CI)	P value	β (95% CI)	P value	β (95% CI)	P value
**HIV RNA**						
Unadjusted model	**-0.08 (-0.16 to -0.00)**	**0.045**	0.06 (-0.07 to 0.20)	0.346	-0.04 (-0.23 to 0.16)	0.691
Adjusted model	-0.08 (-0.16 to 0.00)	0.063	0.06 (-0.07 to 0.20)	0.354	-0.04 (-0.23 to 0.15)	0.672
**CD4 cell count**						
Unadjusted model	0.12 (-0.04 to 0.28)	0.141	-0.02 (-0.27 to 0.22)	0.854	0.13 (-0.22 to 0.48)	0.453
Adjusted model	0.10 (-0.08 to 0.28)	0.261	-0.03 (-0.28 to 0.22)	0.801	0.21 (-0.14 to 0.57)	0.238
**Total bilirubin**						
Unadjusted model	0.03 (-0.66 to 0.71)	0.941	**1.08 (0.22 to 1.94)**	**0.015**	-0.05 (-1.31 to 1.20)	0.931
Adjusted model	0.00 (-0.69 to 0.70)	0.993	**1.09 (0.18 to 2.00)**	**0.019**	-0.11 (-1.37 to 1.16)	0.867
**Fasting glucose**						
Unadjusted model	0.16 (-0.94 to 1.27)	0.772	-0.11 (-1.87 to 1.65)	0.902	-0.92 (-2.91 to 1.06)	0.350
Adjusted model	0.49 (-0.76 to 1.73)	0.439	-0.02 (-2.04 to 2.00)	0.985	-1.87 (-4.04 to 0.31)	0.090
**Total cholesterol**						
Unadjusted model	0.00 (-0.00 to 0.00)	0.679	0.00 (-0.00 to 0.01)	0.611	0.00 (-0.00 to 0.01)	0.292
Adjusted model	0.00 (-0.00 to 0.00)	0.698	0.00 (-0.01 to 0.01)	0.621	0.00 (-0.00 to 0.01)	0.356
**HDL cholesterol**						
Unadjusted model	0.00 (-0.01 to 0.01)	0.549	-0.00 (-0.03 to 0.02)	0.710	0.02 (-0.00 to 0.05)	0.059
Adjusted model	0.00 (-0.01 to 0.02)	0.491	-0.00 (-0.03 to 0.02)	0.702	**0.03 (0.00 to 0.05)**	**0.036**
**LDL cholesterol**						
Unadjusted model	0.00 (-0.00 to 0.01)	0.458	0.00 (-0.01 to 0.01)	0.534	0.00 (-0.01 to 0.01)	0.632
Adjusted model	0.00 (-0.00 to 0.01)	0.479	0.00 (-0.01 to 0.01)	0.540	0.00 (-0.01 to 0.01)	0.660
**Triglycerides**						
Unadjusted model	-0.19 (-0.50 to 0.12)	0.238	0.16 (-0.48 to 0.79)	0.616	-0.58 (-1.31 to 0.16)	0.120
Adjusted model	-0.20 (-0.52 to 0.12)	0.210	0.15 (-0.51 to 0.82)	0.645	-0.60 (-1.34 to 0.15)	0.112
** C-reactive protein**						
Unadjusted model	-0.07 (-0.23 to 0.09)	0.371	0.14 (-0.06 to 0.33)	0.158	-0.05 (-0.33 to 0.23)	0.720
Adjusted model	-0.08 (-0.24 to 0.08)	0.303	0.13 (-0.08 to 0.33)	0.216	-0.02 (-0.31 to 0.28)	0.913

Linear regression models of the association between variables at baseline (HIV RNA, CD4 cell count, total bilirubin, fasting glucose, total, HDL and LDL cholesterols, triglycerides, and c-reactive protein) and hepatic fibrosis scores (FIB-4, BARD and NFS). HIV RNA, CD4 cell count, fasting glucose, triglycerides, and c-reactive protein were log-transformed. Statistical significance was set for a value of p < 0.05

Abbreviations: FIB-4, fibrosis-4; HDL, high-density lipoprotein; HIV, human immunodeficiency virus; LDL, low-density lipoprotein; NFS, NAFLD fibrosis score; RNA, ribonucleic acid

A significant positive association was observed between total bilirubin at baseline and BARD score change (β = 1.09 [0.18 to 2.00]; p = 0.019 in the adjusted model), suggesting that higher baseline bilirubin is associated with an increase in BARD.

The unadjusted linear regression model showed no association between HDL and NFS change, but, when adjusted for age and sex, there was a significant positive association with NFS change (β = 0.03 [0.00 to 0.05]; p = 0.036), indicating that higher baseline HDL cholesterol is associated with an increase in NFS.

No associations were found between any of the fibrosis scores and CD4 cell count, fasting glucose, total and LDL cholesterol, TG and CRP.

## Discussion

In our single-center retrospective assessment of previously naïve persons with HIV on an INSTI based regimen, we observed a significant decrease in the values of FIB-4 and NFS scores, which may indicate a reduction in the risk of developing fibrosis in these patients. Also, we found a significant negative association between HIV RNA load at baseline and FIB-4 variation between baseline and 12 months, suggesting higher HIV RNA at baseline was significantly associated with a greater decrease in FIB-4.

However, we did not find differences in the proportion of individuals in each score category between the first and the last visit, which may be due to the small sample of this study.

Although, we did not see any significant changes in the HSI, that would indicate a change in steatosis, our findings supported that NAFLD is highly prevalent in persons with HIV, as demonstrated in previous studies [4].

Macias et al. compared persons with HIV with NALFD who switched from efavirenz to raltegravir (RAL) with patients maintaining efavirenz-based therapy. After 48 weeks, they found that the patients who switched to RAL showed a reduction in the degree of hepatic steatosis, as measured by Controlled Attenuation Parameter (CAP) as well as a greater proportion of patients without significant steatosis [22]. This study agrees with our findings in suggesting that INSTIs do not contribute to the progression of hepatic steatosis. However, we did not find a similar reduction in hepatic steatosis. The mentioned study measures hepatic steatosis using CAP, a much more sensitive method of evaluating this parameter when compared to the HSI score used in our study, which might explain the differences in results.

On the other hand, Bischoff et al. showed that patients receiving INSTIs had a greater development and progression of steatosis and evolution towards NASH, in relation to increased body weight gain, which is contrary to our findings [10]. Similarly, a prospective cohort study showed that INSTIs were related to greater odds of moderate-to-severe hepatic steatosis. However, they did not find this relation to be true for every INSTI. This association was present for exposure to elvitegravir and RAL, but not to dolutegravir (DTG), even though the patients receiving DTG had the highest weight gain [23].

In our study, the INSTI 96% of patients was receiving was DTG. This way, the previously mentioned study comes to support our findings, and propose a hypothesis as to why they are not congruent with previous studies, such as the one performed by Bischoff et al., in which INSTIs used are not specified. Although INSTIs appear to contribute to the progression of hepatic steatosis in persons with HIV, this might not be true for DTG, despite its effect on weight gain. Riebensahm et al. suggested the same explanation for their findings of lack of relation between INSTIs and hepatic steatosis [8]. Therefore, to support this claim, more studies comparing the various INSTIs and their individual effects on hepatic steatosis are needed.

The patients in the present study showed a significant increase in BMI, which could be explained by multiple factors. On the one hand, several studies demonstrated a greater weight gain in patients receiving INSTI based regimens, especially DTG and RAL, both in ART-naïve and ART-experienced patients [24, 25]. On the other hand, studies have shown that the initiation of ART in treatment-naïve persons with HIV is associated with a short period of weight gain. Considering this is true particularly in patients with lower baseline CD4 + T-cell count and higher HIV RNA viral load, this is consistent with a “return to health effect” [26, 27].

Contrary to the significant decrease in values of FIB-4 and NFS scores, we observed a significant increase in BARD score. These first two scores are continuous variables and BARD score is an ordinal variable, obtained from an addition of points. Although BARD score showed a significant increase, 80% of patients had the same BARD score at baseline and at the last visit, meaning differences were only visible in 13 patients out of 59 in total. Since the calculation of this score includes only BMI, AST/ALT ratio and the presence of diabetes, the fact that BMI showed a significant increase might have had a great impact in BARD score, possibly explaining its elevation. Such an impact would not be so visible in the other scores, since FIB-4 does not include BMI in its calculation and NFS is a much more complex index with various other liver function parameters. Additionally, McPherson et al. compared multiple simple non-invasive fibrosis scoring systems, including the three scores we used in our study, and found FIB-4 score to have the best diagnostic accuracy for advanced fibrosis, with an Area Under Receiver Operator Characteristic Curve (AUROC) of 0.86. The AUROC for NFS was 0.81 and 0.77 for BARD [28]. Imajo et al. compared elastography and various risk scores to histology and found NFS and FIB-4 to be better than other indexes, including BARD, in predicting advanced fibrosis in patients with NAFLD [29]. Accordingly, both the guidelines by the European Association for the Study of the Liver and by the American Association for the Study of the Liver Diseases advocated the use of FIB-4 and NFS to rule out advanced liver fibrosis [3, 13].

The decrease we observed in the risk of developing liver fibrosis, as demonstrated by the reduction in NFS and FIB-4 values, can probably be explained by the effects of ART in the suppression of HIV infection.

HIV infection alone contributes to the development of liver fibrosis, through multiple processes, such as mitochondrial injury, oxidative stress, fatty acid accumulation, gut microbial translocation and immune-activation and proapoptotic effects on hepatocytes [30, 31]. With viral suppression from ART, these mechanisms are reduced, thus decreasing hepatic fibrosis markers and scores in the patients receiving treatment.

Our linear regression model supported this hypothesis by showing that higher HIV RNA at baseline was significantly associated with a greater decrease in FIB-4. This indicates that patients with a higher activity of HIV at baseline, and consequently more liver damage induced by the above-mentioned mechanisms, had a greater reduction in risk of fibrosis with the initiation of treatment. Therefore, these findings support the early initiation of ART.

Multiple previous studies come to support our conclusions, showing that effective ART and complete suppression of HIV replication prevents liver fibrosis development and that modern ART regimens have a negligible effect in its progression [32]. In addition, Blackard et al. found an association between plasma HIV RNA loads and increased FIB-4 in women with HIV with no ART or alcohol use, as well as a negative association between CD4 cell count and FIB-4 [33].

This was also true in HIV-coinfected patients, as shown by Bräu et al., who demonstrated that HIV suppression with ART led to a slower progression rate of HCV-induced fibrosis [34], and by Yang et al. who associated ART initiation with a significant reduction in fibrosis scores in HIV/HBV coinfected patients [35].

Therefore, our findings are more congruent with the effects of ART on viral suppression and may not give us a clear picture of its direct impact in hepatic fibrosis, suggesting the need for future studies in virologically suppressed persons with HIV who switch to INSTIs.

Additionally, the findings of our linear regression model suggested that higher baseline bilirubin is associated with an increase in BARD, which is in line with previous studies that associate advanced liver fibrosis with increased bilirrubin [36]. Furthermore, this model, when adjusted for age and sex, suggested that higher baseline HDL cholesterol is associated with an increase in NFS, which is contrary to what has been shown in prior studies that associate HDL to regeneration and suppression of liver fibrosis [37].

It has been demonstrated that HIV infection is associated with low levels of HDL and that these levels relate to HIV RNA [38, 39]. When further exploring our database, we also found that lower levels of HDL at baseline were related to higher HIV RNA loads. Therefore, a possible explanation for our model findings might be that the patients with higher HDL at baseline had a lower activity of the infection, thus having less effect of the HIV virus in the liver. Consequently, these patients might have had less benefit with the viral suppression exerted by the initiation of ART, showing no reduction in the fibrosis scores. The association with an increase in NFS can then be explained by the increase shown in BMI.

Our study had several limitations. It was a retrospective assessment of a small predominantly male cohort from one center in the north of Portugal, with no control group, therefore the results may not be generalizable to other populations. Our short follow-up time of 12 months allows us only to evaluate the short-term impact of the INSTIs and may underestimate their effect on liver steatosis and fibrosis on the long run. We used serum biomarkers to evaluate the presence of steatosis and fibrosis that have lower sensitivity and specificity than the gold standard test, liver biopsy, and we did not exclude patients with other liver diseases. Other limitations were present in the availability of patient’s data, possibly due to the COVID-19 period and the use of telephonic or virtual consultations. Weight and height information were not available for every patient at the three evaluation times, which led to BMI calculation only being possible in 59 patients. Additionally, only self-reported, not quantitatively specified, alcohol consumption was available, which might have led us to underestimate the presence of alcohol consumption in a small percentage of patients. Furthermore, data on waist and hip circumferences were not available. Consequently, we evaluated weight gain only considering BMI, which does not give information regarding the distribution of fat and presence of visceral fat, important factors in NAFLD.

## Conclusion

In this monocenter cohort of persons with HIV, INSTIs had no impact on hepatic steatosis, mainly driven by the use of a DTG-containing regimen. Additionally, INSTIs were associated with a significant increase in BMI, that might be explained by the direct effect of DTG and/or by the “return-to-health effect” observed with ART initiation. Furthermore, INSTIs were associated with a reduction in the risk of liver fibrosis in persons with HIV, probably due to their effect on suppression of viral replication, perhaps demonstrating a protective action against fibrosis progression.

Therefore, our study highlights the need for early initiation of ART, namely INSTI, as well as a close monitorization of patients with NAFLD, a disease with high prevalence among persons with HIV, in order to prevent the progression towards NASH and liver fibrosis.

### Electronic supplementary material

Below is the link to the electronic supplementary material.

**Additional file 1**: Comparison of baseline characteristics of cohort with and without all liver scores available at baseline

## Data Availability

The datasets used and/or analysed during the current study are available from the corresponding author on reasonable request.

## Declarations

The requirement for a signed informed consent was waived by Comissão de Ética para a Saúde do Centro Hospitalar Universitário de São João.

This study was performed in accordance with the Declaration of Helsinki, the world Health Organization, the Oviedo Convention and the ICH guideline for good clinical practice.

## Abbreviations

AIDS: Acquired Immunodeficiency Syndrome
ALT: Alanine Transaminase
AST: Aspartate Transaminase
AUROC: Area Under Receiver Operator Characteristic Curve
BMI: Body Mass Index
CAP: controlled attenuation parameter
ART: combined Antiretroviral Therapy
CDC: Centers for Disease Control and Prevention
CRP: C Reactive Protein
DTG: Dolutegravir
EASL-EASD-EASO: European Association for the Study of the Liver-European Association for the Study of Diabetes-European Association for the Study of Obesity
ELF: Enhanced Liver Fibrosis
FIB-4: Fibrosis-4 Index
HBV: Hepatitis B Virus
HCV: Hepatitis C Virus
HDL: High-density Lipoprotein
HIV: Human Immunodeficiency Virus
HSI: Hepatic Steatosis Index
INSTIs: Integrase Strand Transfer Inhibitors
LDL: Low-density Lipoprotein
NAFL: Non-alcoholic fatty liver
NAFLD: Non-alcoholic Fatty Liver Disease
NASH: Non-alcoholic Steatohepatitis
NFS: NAFLD Fibrosis Score
NNRTIs: Non-Nucleoside Reverse-Transcriptase Inhibitors
NRTIs: Nucleoside/nucleotide Reverse-Transcriptase Inhibitors
PIs: HIV protease inhibitors
RAL: Raltegravir
RNA: Ribonucleic Acid
T0: Before starting ART
T12: Twelve months of follow-up
T6: Six months of follow-up
TAF: Tenofovir-alafenamid
TG: Triglycerides
