# Supplementary material for: The impact of integrase inhibitors on steatosis and fibrosis biomarkers in persons with HIV naïve to antiretroviral therapy
Source: BMC Infect Dis. 2023 Aug 24;23:553. doi: 10.1186/s12879-023-08530-3 (PMC10464196; doi:10.1186/s12879-023-08530-3)
Supplement: Supplementary file 1 — Additional file 1: Comparison of baseline characteristics of cohort with and without all liver scores available at baseline [file 12879_2023_8530_MOESM1_ESM.pdf]

Additional File 1

File name: Additional file 1

File format: .pdf

Title of data: Comparison of baseline characteristics of cohort with and without all liver scores available at baseline

Description of data: We present a Table comparing the baseline characteristics of the sample that has all the hepatic steatosis and fibrosis scores (HSI, FIB-4, NFS and BARD) available in the first visit (n=59) with the baseline characteristics of the individuals without at least one of these scores (n=40). We can see that the characteristics of the individuals are similar between these groups and similar to the whole cohort.

Comparison of baseline characteristics of cohort with and without all liver scores available at baseline

Parameter	Cohort without at least one of the liver scores at baseline (n=40)	Cohort with all liver scores available at baseline (n=59)	P
Male	33 (82.5)	48 (81.4)	0.88
Age, years	41.0 (28.0; 55.5)	35.0 (28.0; 49.0)	0.38
Smoker	17 (45.9)	22 (40.0)	0.57
BMI, kg/m^2	23.73 (4.97)	23.75 (3.57)	0.99
HIV-related parameters			
HIV RNA, 10^4 copies/mL	8.69 (1.96;28.85)	10.30 (3.60;25.10)	0.65
HIV RNA (<50)	0 (0.0)	0 (0.0)	
CD4 cell count, cells/μL	258 (85;543)	261 (114;424)	0.89
HIV risk factor			0.73
Injecting drug user	0 (0.0)	1 (1.8)	
Homosexual contact	24 (66.7)	37 (64.9)	
Heterosexual contact	12 (33.3)	18 (31.6)	

CDC stage			0.72
A	24 (60.0)	37 (62.7)	
B	10 (25.0)	11 (18.6)	
C	6 (15.0)	11 (18.6)	
ART Regimen			0.32
TDF/FTC + DTG	18 (45.0)	18 (30.5)	
ABC/3TC/DTG	11 (27.5)	25 (42.4)	
3TC/DTG	9 (22.5)	13 (22.0)	
FTC/TAF/BIC	1 (2.5)	3 (5.1)	
TAF/FTC + DTG	1 (2.5)	0 (0.0)	
Analytical parameters			
Fasting Plasma Glucose, mg/dL	88.00 (82.00;93.00)	87.00 (80.00;94.00)	0.68
Triglycerides, mg/dl	100.00 (66.00;120.00)	95.00 (74.00;131.00)	0.94
Total cholesterol, mg/dl	156.40 (41.17)	157.04 (45.74)	0.95
HDL, mg/dl	40.00 (13.13)	40.38 (12.84)	0.90
LDL, mg/dl	99.63 (30.57)	101.67 (32.97)	0.80
AST, U/L	28.00 (19.00;39.00)	26.00 (22.00;31.00)	0.42
ALT, U/L	21.00 (13.00;49.00)	23.00 (15.00;32.00)	0.99
Total bilirubin, mg/dL	0.59 (0.16)	0.61 (0.25)	0.71
Platelets, 10^3/μL	195.28 (80.95)	217.42 (76.18)	0.17
Albumin, g/L	40.84 (5.87)	38.84 (7.88)	0.22
Creatinine, mg/dL	0.79 (0.67;0.88)	0.76 (0.65;0.90)	0.52
Uric acid, mg/dL	5.90 (4.30;6.20)	5.45 (4.90;6.50)	0.94
CRP, mg/L	3.65 (2.00;16.60)	4.70 (1.50;25.90)	0.82

Data are shown as mean (standard deviation), median (interquartile range) or n (%). P values were obtained using independent samples t-test, Mann-Whitney U or chi-square test where appropriate. Abbreviations: 3TC, lamivudine; ABC, Abacavir, ALT, alanine aminotransferase; AST, aspartate aminotransferase; BIC, bictegravir; BMI, body mass index; CRP, C-reactive protein; DTG, dolutegravir; FIB-4, fibrosis-4; FTC, emtricitabine; HDL, high-density lipoprotein; HSI, hepatic steatosis score; HIV, human immunodeficiency virus; LDL, low-density lipoprotein; NFS, NAFLD fibrosis score; RNA, ribonucleic acid; TAF, tenofovir alafenamide; TDF, tenofovir disoproxil fumarate.
